# TMBserval: a statistical explainable learning model reveals weighted tumor mutation burden better categorizing therapeutic benefits

**DOI:** 10.3389/fimmu.2023.1151755

**Published:** 2023-05-10

**Authors:** Yixuan Wang, Jiayin Wang, Wenfeng Fang, Xiao Xiao, Quan Wang, Jian Zhao, Jingjing Liu, Shuanying Yang, Yuqian Liu, Xin Lai, Xiaofeng Song

**Affiliations:** ^1^ Department of Biomedical Engineering, College of Automation Engineering, Nanjing University of Aeronautics and Astronautics, Nanjing, China; ^2^ School of Computer Science and Technology, Faculty of Electronics and Information Engineering, Xi’an Jiaotong University, Xi’an, China; ^3^ State Key Laboratory of Oncology in South China, Collaborative Innovation Center for Cancer Medicine, Sun Yat-sen University Cancer Center, Guangzhou, China; ^4^ Genomics Institute, Geneplus-Shenzhen, Shenzhen, China; ^5^ Department of Respiratory and Critical Care Medicine, The Second Affiliated Hospital of Xi’an Jiaotong University, Xi’an, China

**Keywords:** clinical immunology, multidimensional tumor mutation burden, multiple instance learning, categorical decision-making, statistical interpretability, model calibration

## Abstract

A high tumor mutation burden (TMB) is known to drive the response to immune checkpoint inhibitors (ICI) and is associated with favorable prognoses. However, because it is a one-dimensional numerical representation of non-synonymous genetic alterations, TMB suffers from clinical challenges due to its equal quantification. Since not all mutations elicit the same antitumor rejection, the effect on immunity of neoantigens encoded by different types or locations of somatic mutations may vary. In addition, other typical genomic features, including complex structural variants, are not captured by the conventional TMB metric. Given the diversity of cancer subtypes and the complexity of treatment regimens, this paper proposes that tumor mutations capable of causing various degrees of immunogenicity should be calculated separately. TMB should therefore, be segmented into more exact, higher dimensional feature vectors to exhaustively measure the foreignness of tumors. We systematically reviewed patients’ multifaceted efficacy based on a refined TMB metric, investigated the association between multidimensional mutations and integrative immunotherapy outcomes, and developed a convergent categorical decision-making framework, TMBserval (Statistical Explainable machine learning with Regression-based VALidation). TMBserval integrates a multiple-instance learning concept with statistics to create a statistically interpretable model that addresses the broad interdependencies between multidimensional mutation burdens and decision endpoints. TMBserval is a pan-cancer-oriented many-to-many nonlinear regression model with discrimination and calibration power. Simulations and experimental analyses using data from 137 actual patients both demonstrated that our method could discriminate between patient groups in a high-dimensional feature space, thereby rationally expanding the beneficiary population of immunotherapy.

## 1 Introduction

Tumor mutation burden (TMB), typically reported as the number of non-synonymous mutations per mega-base (1–4), has a positive probabilistic link with the neoantigens that embody the antitumor rejection (5–7). TMB-high is widely recognized as a clinically available biomarker, driving sustainable responses to immune checkpoint inhibitors (ICI) and being implicated in better prognosis (8, 9). However, the fact that it is a one-dimensional numeric representation has attracted criticism, since it provides a limited portrayal of genetic potential (10). Single nucleotide variant (SNV) and insertion/deletion (Indel) are treated identically by currently accepted TMB assays, with equal weighting given to each mutation. A numerical index that merely counts the overall number of sequence alternations may be insufficient for steering immunotherapy decisions, since many studies have shown that different genomic mutations trigger different immunotherapeutic responses. For example, it has been demonstrated that Indel-derived neoantigens are highly immunogenic and enriched in mutant-binding specificity relative to SNV-derived neoantigens (11); this in intuitive, since even a minor Indel can cause a frameshift variant, yielding additional neopeptides or neoepitopes. In addition, somatic mutations in various DNA repair pathways or clonal structures can also cause the generation of diverse neoantigens with unique antitumor immune functions (12–14). Niknafs et al. (15) further revealed that mutations located in single-copy regions or present in multiple copies in the cancer genome—which are unlikely to be lost—are referred to as persistent TMB and act as intrinsic drivers of sustained immunological tumor control. Other potential sources of neoantigens, such as complex structural alterations, are also not captured by TMB. Consequently, the TMB index cannot adequately explain the manifestation of neoantigens.

Given the narrow representativeness, researchers have suggested separate counts of genomic mutations that are indicative of distinct forms of antitumor immunogenicity (10). Thus, the one-dimensional index must be partitioned into vectors with higher dimensions. Stratification of clinical cohorts is facilitated by a systematic examination of immunotherapy outcomes based on refined TMB vectors, yielding a convergent decision-making framework from multidimensional mutations to multifactorial efficacy. However, extant statistical models cannot satisfy the requirements for fusion modeling due to the consequent computational challenges. The mutual exclusion and co-occurrence of multidimensional mutations must be considered first (16–18). Meanwhile, the clinical endpoints of immuno-oncology are often complicated and varied (19, 20). They typically include measures such as the objective response rate (ORR) and time-to-event (TTE), and TMB used to predict both (21). Diverse decision endpoints may be potentially interdependent due to patient homology, where associations fluctuate with cancer species and therapeutic regimens (22–24). The intricate reliance between multidimensional mutant targets and the relationship between decision endpoints is a key computational hurdle. In addition, existing mathematical methods struggle to overcome computational constraints imposed by the nonlinear association between mutations and efficacy, especially for high-dimension datasets. For the joint model previously proposed in TMBcat (25, 26), input modeling remained linear in functional form, and thus this model experienced information loss during the capture of nonlinear information. However, establishing a coupled nonlinear regression model between high-dimensional inputs and outputs will significantly strain model parameter estimation. Thus, the TMBcat formula, together with its explicit analytical solutions, will no longer be applicable, and will result in prohibitive computational time and cost expenses to obtain the first-order derivatives of the likelihood function and the information matrix. This will make the process unaffordable for practical applications. Furthermore, conventional statistical methods are model-driven, meaning that their structure must be specified *a priori* using empirical or analytical approaches (27). Improper model assumptions can pose an unacceptable risk of misspecification. Traditional numerical approaches therefore make it challenging to establish reasonable, many-to-many, nonlinear regression models for non-independent high-dimensional input covariates and non-independent dependent variables.

**Figure 1:**
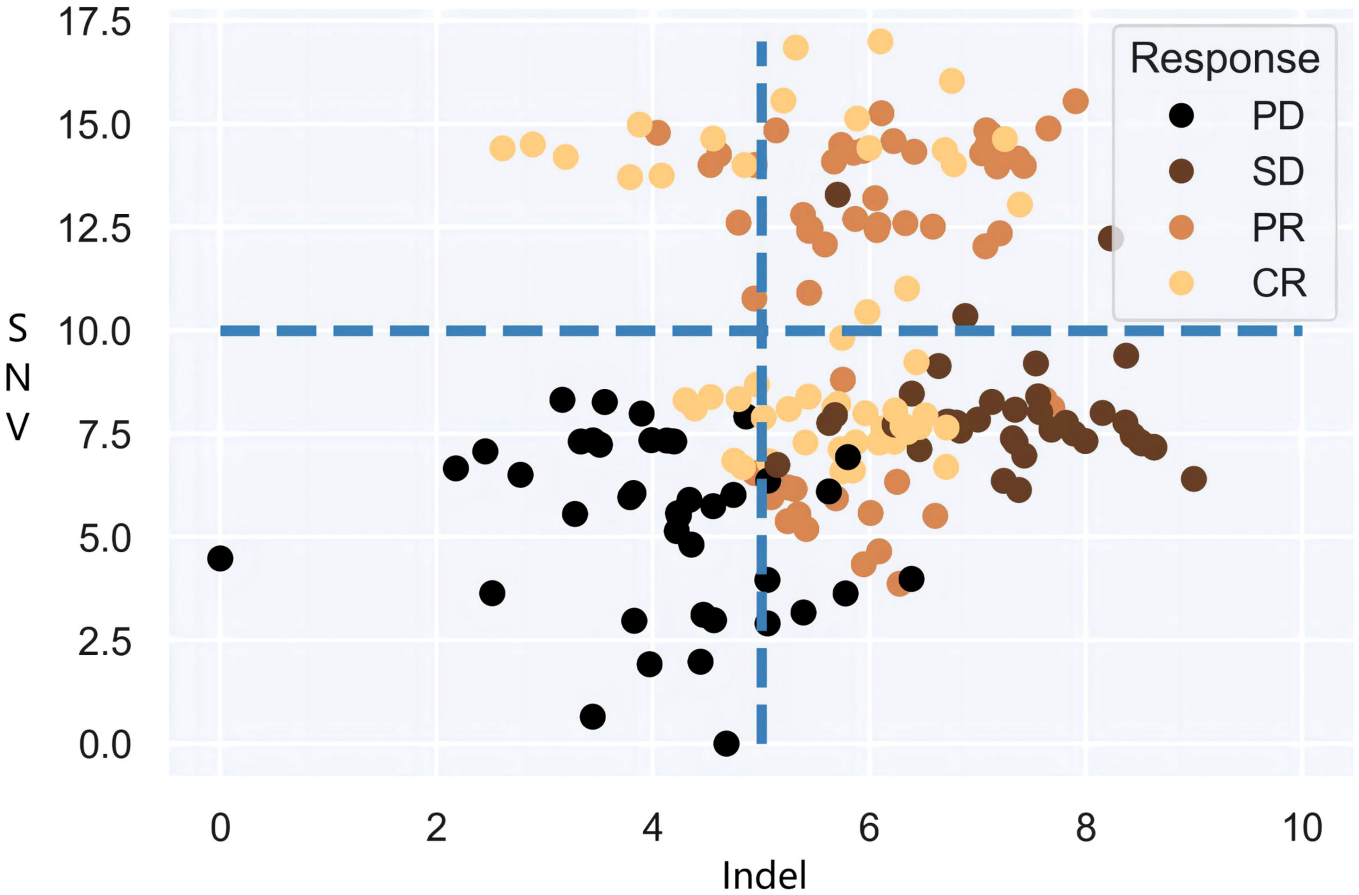
Differentiation of SNV and Indel burden for patient tumor response.

It is also noteworthy that the objective of clinical practice is effective categorical decision-making, and there is an urgent need of a definitive and impeccable decision-supporting criterion based on dependable predictive biomarkers, i.e., joint multi-categorization thresholds. Therefore, the focus of this paper is to investigate the categorization boundaries that form authentic patient subgroups for immunotherapy cohorts in large populations and to establish a map of high-dimensional categorization thresholds for patient subgroups. Undoubtedly, statistical models will confront the combinatorial explosions that stem from threshold demarcation of high-dimensional mutational burdens. Models can dichotomize or multi-categorize patients based on single decision targets if given specific decision criteria. Yet when more than one decision biomarker is accessible, the intersection and/or union of multiple burdens cannot be carried out immediately. As shown in **Figure 1**, the fixed cut-offs of two mutation burdens are somewhat arbitrary, and neither intersection nor union can optimally distinguish between responders and non-responders. It goes without saying that as more biomarkers are included, the number of patient subgroup combinations grows factorially, a rate that standard models cannot handle effectively. Therefore, exploring the underlying association between high-dimensional mutational burdens and ICI presents a key technical challenge.

In summary, here we attempt to build a pan-cancer-oriented many-to-many nonlinear regression model to rationally map synergistic mutational burdens to fusion endpoints for the rapid prediction and classification of unseen patients or patient subgroups. To solve the technical challenges discussed above, we propose a convergent categorical decision-making framework called TMBserval (‘Statistical Explainable machine learning with Regression-based VALidation’). On one hand, TMBserval leverages machine learning techniques, such as artificial neural networks (ANN) to map from multidimensional mutations to multiscale endpoints, thereby addressing broad associations. It also uses multiple-instance learning (MIL) concepts to build a prognostic prediction model based on patient subgroups as opposed to individuals, thereby facilitating categorical decision-making. On the other hand, TMBserval also uses standard statistical criteria for discrimination and calibration measures for the learning phase, which renders the trained model more statistically interpretable and removes the black-box phenomenon that is sometimes characteristic of machine learning. This makes our model more accessible to clinicians. To verify the predictive capacity of TMBserval, we conducted a series of simulation experiments; these results supported the superiority of TMBserval’s predictions for unseen patient/patient groups. We then gathered a cohort of 73 patients with non-small-cell lung cancer (NSCLC) and 64 patients with nasopharyngeal carcinoma (NPC) who were treated at the Sun Yat-sen University Cancer Center (SYUCC). The results of the cohort dataset further demonstrate the applicability of the proposed model to clinical practice. Taken together, TMBserval can achieve the optimization goal of rationally categorizing patients and refining differences among patient subgroups even in a high-dimensional feature space. The source code to reproduce our results can be downloaded from https://github.com/YixuanWang1120/TMBserval.

## 2 Materials and methods

This paper aims to establish a reasonable many-to-many mapping relationship between non-independent input mutations and non-independent output endpoints, thereby resulting in proper categorization of ICI patients. In conventional statistical regression, complex interdependencies across mutational variables and relationships between decision endpoints can lead to multicollinearity (28). In regression models showing high multicollinearity, explanatory variables may exhibit convergence. This means that different inputs may contribute to the same variation in the dependent variable (29), making it difficult to assess the effects of different independent variables and causing the estimates of regression coefficients to no longer be valid and unbiased. Furthermore, parameter variance estimates are proportional to the inverse of the explanatory variables, and collinearity can cause the matrix to converge to zero, thus amplifying the magnitude of the variances. In this case, small perturbations in the sample can trigger large fluctuations in the estimates, causing model instability and generalization errors. In standard statistical analysis, Ridge or Lasso regression is frequently used to solve this problem (30). However, these strategies produce fundamentally biased estimators, as they seek equations with more realistic estimated coefficients that are slightly less effective and poorly interpretable. In contrast, oncology trials are explicitly intended to investigate the association between mutational effects and treatment outcomes; they therefore substantially rely on coefficient unbiasedness to a greater degree than would otherwise be the case.

Therefore, we consider the integration of machine learning concepts with statistical approaches to address modeling limitations in fusion decision-making. ANNs, as data-driven methods, can include infinitely approximate nonlinear functions of arbitrary form due to their nonparametric nature. Meanwhile, the collinearity between mutations can be discerned within the network (i.e., as similar weights between the input neurons and hidden layers) without affecting prediction results and while remaining free from the ill-conditioned problem that affects traditional regression modeling (31). Thus, the modeling intention of this paper is to obtain exhaustive and generalizable categorization criteria for immunotherapy patients, thereby identifying associations between mutational features that fall within a particular threshold boundary and in a specific patient group. Here we use multiple-instance learning (MIL) to construct a prognostic prediction model based on patient subgroups instead of individuals. This is not because individual labels are uncertain but instead due to the necessity of establishing a broad categorization standard. Also, information about individual patients is not simply discarded but described by aggregated subgroup-level features.

### 2.1 Model specification

The multi-biomarker-multi-endpoint regression model implemented using MIL concept is defined as follows.

Suppose a given training set consists of a total of *N* patients, forming *M* patient subgroups, i.e., *M* bags $\left\{G_{1},G_{2}\ldots G_{M}\right\}$ , with subgroup *m* consisting of *n_m_* patients. Thus $G_{m}=\{x_{m1},x_{m2},\ldots ,x_{mn_{m}}\}$ , $\sum_{m=1}^{M}n_{m}=N$ . Any individual patient’s feature vector consists of multidimensional genomic mutations capable of triggering different degrees of antitumor immunogenicity. In that case, each patient $x_{mi}\;(i=1,\ldots ,n_{m})$ is represented by a *d*-dimensional genomic mutation vector, and the *i*th patient of the *m*th subgroup can be represented as: ${\left[x_{m1},x_{m2},\ldots ,x_{mid}\right]}^{T}$ . Formally, a patient *x* characterized by a *d*-dimensional mutational vector corresponds to a point in the instance space 𝕏 $\subseteq {\mathbb{R}}^{d}$ , while subgroup *G* consists of numerous patients in this space, i.e.,*G* = {*x*_*i*_ ∈ 𝕏 | *i* ∈ { 1, ...,*n*}}. We further assume that each patient has a prognostic label of *y_x_*. However, even in the training set, these individual-level labels are unknown, and we observe only the prognostic assessment results *y* for different patient subgroups. MIL therefore provides theoretical support to realize the subgroup-based training purpose of this paper.

The concept of MIL was first introduced in 1997 by Dietterich et al. (32) to predict drug activity. The training set consists of several bags with labels; each bag contains several instances with unknown labels. If at least one instance in a specific bag is a positive example, it is labeled positively; otherwise, it is labeled negatively. Therefore, the package is labeled as $y=\underset{x\in X}{{\max y}_{x}}$ . In contrast, in our ICI categorical decision-making model, the prognosis of each subgroup is determined by the performance of the patient groups it comprises and is evaluated using a variety of methods that correspond to different clinical judgment criteria. The specific subgroup label definitions that we consider for statistical interpretability are described in detail in a subsequent subsection.

The task of MIL is to perform concept learning based on bags with known labels in a training set to correctly label unseen bags (33). Similarly, here we use a neural network (NN) with *d* input units and one output unit for concept learning to properly identify unseen patients/subgroups by using patient subgroups with known labels in a training dataset. The structure of the NN can be abstracted as an objective function f:x→y . The optimization goal is to establish a reasonable, many-to-one, or many-to-many, nonlinear map between the feature vector ${\left[x_{m1},x_{m2},\ldots ,x_{mid}\right]}^{T}$ of patient subgroups $G_{m}=\{x_{m1},x_{m2},\ldots ,x_{mn_{m}}\}$ and their prognostic label *y_m_* by minimizing the loss between the actual and desired outputs. If successful, this would ensure that for any new patients or subgroups, their labels can be accurately predicted. In general, learning using supervised NN learners focuses on predicting patient prognosis since all training instances are labeled under supervision; this makes attaining the learning goal feasible. However, for our categorical decision-making framework, the emphasis on learning shifts from identifying patients to distinguishing among subgroups. This is because the overall prognosis of patients within a subgroup determines the output label of that subgroup. Hence, the specific loss function for the training process of TMBserval must be defined distinctly.

### 2.2 Discrimination and calibration amelioration

Typically, the evaluation of regression model performance involves considering both discrimination and calibration power. Discrimination is the capacity of a model to accurately classify a cohort of patients as superior or inferior or to identify individuals as low risk or high risk. Calibration, on the other hand, refers to the consistency between the likelihood of an outcome occurring and its probability as predicted by the model; hence, this is also referred to as consistency or goodness-of-fit. The former reflects whether the model is capable of discriminating for the patient cohort, while the latter reflects the accuracy with which a model predicts absolute risk. For the proposed TMBserval, the discriminative power of the model is captured by the ability of the prognostic labels used to discriminate within the patient cohort, while the loss metric function is responsible for optimizing model calibration.

With respect to discrimination, we obtained subgroup-level labels by using the following approach. First, for the multi-biomarker-multi-endpoint regression model developed here, repeatedly building joint regression models for prognostic labels suffers from information redundancy. As stated previously, the joint statistical model cannot be applied to the investigation of multidimensional mutation burdens. However, repeatedly building regression models can cause covariates with significant effects to dominate the analysis, thereby weakening the effects of the remaining covariates on regression endpoints, which can lead to biased inferences. To fix this, multiple methods of labeling patient subgroup prognostic labels by considering discrimination factors from the following two perspectives were implemented to optimize our model.

#### 2.2.1 Specialist labeling based on prognostic categories

Clinicians typically employ this label to categorize cohort patients with a specific cancer type and treatment regimen based on their clinical experience. Thus, patients can be assigned to different categories (e.g., “effective/ineffective” or “good/moderate/poor” prognosis) according to the performance of different patient subgroups with respect to tumor remission and survival. More specific differentiation criteria can include tumor status, which is generally classified as complete response (CR), partial response (PR), stable disease (SD), and progressive disease (PD) according to the Response Evaluation Criteria in Solid Tumors [RECIST version 1.1 (34)]. Patients with CR or PR are usually labeled as treatment-effective controls. Moreover, judging TTE benefits after treatment, including the time from initial dosing to PD or death from any cause is referred to as progression-free survival (PFS), and the time from the first dose to death is referred to as overall survival (OS). In general, a PFS of more than 6 months is deemed to be a favorable prognosis. Thus, when we must analyze treatment performance by combining these two efficacy endpoints, we can refer to the joint model proposed by TMBcat to determine the probability of joint benefits for patients, as well as the between-group discrepancy maximization method used to group patients (25, 26). In this case, label ∈ {0,1} or label ∈ {−1,0,1} . This form of labeling is straightforward, can be easily learned by machine learning models, and is simple to understand and implement in clinical practice. The disadvantage, however, is that this kind of data labeling is highly subjective and dependent on expert experience, which has some unreasonableness and restrictions.

#### 2.2.2 Prognostic probability labeling based on objective patient endpoints

Since manual prognostic category labeling suffers from excessive subjectivity, we also explored labeling based on objective observations extracted from patient clinical records. For each patient subgroup’s prognostic label, we integrate the ORR and TTE endpoints to thoroughly evaluate efficacy. The ORR endpoint is the proportion of tumor responses in a patient subgroup that can be used to directly quantify treatment efficacy. Four distinct interpretations based on the median, maximum, minimum, and mean are provided in this paper for various cancer types and ICI treatment regimens on TTE endpoints. This is due to the fact that in clinical scenarios, different definition criteria are chosen for the prognosis definition of a patient subgroup according to different treatment requirements. For example, a clinical trial for a class of drugs can be designed to require a certain level of median survival time for a patient group before the treatment will be deemed effective. Naturally, the prognostic labeling of patient subgroups should also be determined based on the median survival of the patients included, i.e., $label\;={\left[ORR,TTE\_med\right]}^{T}$ .

In addition, ORR and TTE outcomes for malignancies also have distinct data scales and measurement units. In particular, the ORR endpoint, representing the proportion of patients with tumor response in the subgroup, is a scale value between 0 and 1. When the unit of measure of the TTE endpoint is days, the span of the difference in time is significantly larger than the span of the scale value, which causes the distance between samples to be dominated by the TTE endpoint. Similarly, if the unit of measure of the TTE endpoint is changed to years, it may also be difficult for the learner to learn because differences are too large. Therefore, to reflect the importance of each dimensional feature simultaneously, the prognostic labels of the subgroups in this paper are normalized in a probability mapping style. The survival endpoint TTE is modeled using standard Cox PH regression to assess the survival risk of patients given factors related to tumor progression. Consequently, *p_T_* was then generally expressed as the probability of survival beyond a predetermined time point, at which $label=\text{median}{\left[p_{R},p_{T}\right]}^{T}$ .

Next, to establish the loss function, we propose statistically interpretable loss metrics for different types of prognostic labels. First, we employ prognostic categories $y_{m}\in \{0,1\}$ , which are manually labeled. Suppose *o_mi_* is the probability that the network *f* predicts a positive prognostic label for patient *x_mi_*. When research data characterizes prognostic performance based on the median criterion, the output *G_m_* of the network *f* is ${\hat{o}}_{m}=\underset{1\leq i\leq n_{m}}{\text{median}o_{\text{mi}}}$ . This paper proposes that the global calibration loss of the network on the training set is defined as:


(1) $$E=\sum_{m=1}^{M}E_{m}=\sum_{m=1}^{M}\frac{{\left(y_{m}-{\hat{o}}_{m}\right)}^{2}}{{\hat{o}}_{m}}+\frac{{\left(y_{m}-{\hat{o}}_{m}\right)}^{2}}{1-{\hat{o}}_{m}}$$

When the denominator term is eliminated from the loss function, Eq. (1) approximates the mean square error (MSE) metric utilized in machine learning. In addition, in Eq. (1), when patient subgroup efficacy is evaluated entirely using the mean criterion, the optimization objective of the model is equivalent to the Hosmer-Lemeshow goodness-of-fit test, where the calibration loss approximately follows a chi-square distribution with *M*-2 degrees of freedom. Therefore, limiting the loss Eq. (1) not only minimizes the global loss of the training network but also optimizes the model’s goodness-of-fit, thereby making the trained model *f* more relevant and applicable.

However, when the prognostic probability labels $y_{m}=\text{median[}p_{R},p_{T}{]}_{m}^{T}$ for objective patient endpoints are used, the calibration loss between the actual and expected output must be quantified by the distance for vector-based labels. For an actual output *o_mi_* corresponding to patient *x_mi_*, its magnitude is determined by the distance to the spatial origin. Similarly, when this paper describes the prognostic performance of a patient subgroup based on the median criterion, the global error of the network on the training set is then defined as:


(2) $$E=\sum_{m=1}^{M}E_{m}=\sum_{m=1}^{M}dist({\hat{o}}_{m},y_{m})$$

Here, *dist*(·,·) denotes the distance metric formula and ${\hat{o}}_{m}=\underset{1\leq i\leq n_{m}}{\text{median}dist(o_{mi},}0)$ . For the ORR and TTE endpoints, this paper proposes several distance metrics to better quantify their correlational relationships, including the Euclidean, Mahalanobis, and Minkowski distances.

When the correlation between the ORR and TTE endpoints is ignored, or the fluctuation is substantially more minor, we advocate using the standard Euclidean distance:


(3) $$dist(y_{1},y_{2})=\sqrt{(y_{1}-y_{2}){\left(y_{1}-y_{2}\right)}^{T}}$$

The Euclidean distance provides a more intuitive measure of the spatial distance between vectors and is suitable for cases where the weights of the different endpoints are equivalent. When the fluctuation in the correlation between the ORR and TTE endpoint can impact the model inference, the Mahalanobis distance of the covariance matrix should be used instead:


(4) $$dist(y_{1},y_{2})=\sqrt{(y_{1}-y_{2})({\text{V}}^{-1}){\left(y_{1}-y_{2}\right)}^{T}}$$

Here, **V** is the covariance matrix between the ORR and the TTE endpoint, and can be calculated from the observed data of a subgroup of patients. If the covariance matrix is a unit matrix, then the Mahalanobis distance is reduced to the Euclidean distance. The Mahalanobis distance, on the other hand, is scale-independent—i.e., it is independent of the measurement scale and can take into account the connection between the endpoints, excluding the interference of correlation between variables.

When the loss function Eq. (2) is measured using the Euclidean distance, the loss is equivalent to the explained sum of squares used in multivariate statistical analysis. Furthermore, after dividing by the total sum of squares, the optimization objective of the model is equivalent to the coefficient of determination R^2^ in the goodness-of-fit test. Therefore, a network using the loss function Eq. (2) as the optimization objective both minimizes global loss and ensures the goodness-of-fit of the model. making the trained regression model more statistically interpretable.

After an acceptable loss function definition, we employ a back propagation technique based on gradient descent to minimize the loss function *E* and output the optimized objective function. Minimizing global loss is itself a convex optimization problem; therefore, hence a global optimal solution must exist. Since both the Euclidean and Mahalanobis distance formulas can be derived, the gradient descent method based on the loss function *E* is viable.

The specific flow of the fusion decision model as constructed is shown in **Algorithm 1**.

Algorithm 1 Decision-making from multi-biomarker synergy to multi-endpoint fusion.
**Input**: *M* patient subgroups 
$G_{m}=\{x_{m1},x_{m2},\ldots ,x_{mn_{m}}\}$
, with the *m*th subgroup encompassing *n_m_
* patients, where patient *x_mi_
* is characterized by the *d*-dimensional feature vector 
${\left[x_{m1},x_{m2},\ldots ,x_{mid}\right]}^{T}$
.
**Output:** prognosis label 
$\left\{y_{1},y_{2},\ldots ,y_{M}\right\}$
.
1. Based on the given input and output features, build the mapping 
$f:x\in {\mathbb{R}}^{d}\to y$
.
2. Calculate the loss between the actual and desired output *E*.
3. **While** 
E>δ
 **do
If** 
$y_{m}\in \{0,1\}$
 **do
If** criterion=median **do**

$E=\sum_{m=1}^{M}\frac{{\left(y_{m}-{\hat{o}}_{m}\right)}^{2}}{{\hat{o}}_{m}}+\frac{{\left(y_{m}-{\hat{o}}_{m}\right)}^{2}}{1-{\hat{o}}_{m}}$


${\hat{o}}_{m}=\underset{1\leq i\leq n_{m}}{\text{median}o_{mi}}$

**End If
End If
If** 
$y_{m}=\{ORR,TTE\_median^{\prime }\}$
 **do
If** criterion=median **do
If** dist=Euclidean **do**

$E=\sum_{m=1}^{M}\sqrt{(\sqrt{{\hat{o}}_{m}{\hat{o}}_{m}^{T}}-y_{m}){\left(\sqrt{{\hat{o}}_{m}{\hat{o}}_{m}^{T}}-y_{m}\right)}^{T}}$


${\hat{o}}_{m}=\underset{1\leq i\leq n_{m}}{\text{median}o_{mi}}$

**If** dist=Mahalanobis **do**

$E=\sum_{m=1}^{M}\sqrt{(\sqrt{{\hat{o}}_{m}{\text{V}}^{-1}{\hat{o}}_{m}^{T}}-y_{m}){\text{V}}^{-1}{\left(\sqrt{{\hat{o}}_{m}{\text{V}}^{-1}{\hat{o}}_{m}^{T}}-y_{m}\right)}^{T}}$


${\hat{o}}_{m}=\underset{1\leq i\leq n_{m}}{\text{median}o_{mi}}$

**End If
End If
End If**
4. Minimize the loss function *E* by gradient descent.

## 3 Experiments and results

### 3.1 Generation of simulation data

To illustrate the validity of the suggested fusion decision model TMBserval, we simulated an antitumor mechanistic dataset. Data regarding genetic mutations (including SNVs, Indels, and structural variants SVs) and their clinical efficacy endpoints (including ORR and TTE) were simulated for patients receiving immunotherapy with reference to existing public databases of cancer research phenotypes.

We selected the human genomic DNA standard sample NA12878 from the 1000 Genomes Project. Based on a whole exome sequencing (WES) file for chromosome 19, we simulated mutations using BamSurgeon (35). Mutations were simulated at common tumor mutation loci reported in the TCGA database. We focused on immunotherapy-sensitive cancer species, such as NSCLC and breast cancer. WES sequencing data for NA12878 were downloaded in FASTQ format. FASTQ files were processed using Trimmomatic for quality control, where we removed low quality (below 20) or N bases from leading/trailing DNA. After removing splice sequences, correction, and shearing, high-quality paired-end reads were aligned to the human reference genome hs37d5 using the Burrows-Wheeler Aligner. The resulting alignment files (in BAM format) were filtered and cleaned according to the standardized flow recommended by GATK4. This included deduplication, BQSR, and Indel realignment. Filtered BAM files were divided into pairs, with one normal BAM file and one BAM file to be simulated *via* random sampling. ICI-sensitive somatic mutations, including SNVs and Indels, were randomly selected from the TCGA database and added to the simulated BAM file using BamSurgeon.

To simulate the clinical testing process and to train learners to handle measurement errors, we characterized the genomic features of patients based on the type and number of mutations reported by GATK-Mutect, the most commonly used screening software. Mutations were then filtered further using the following criteria: 1) more than 4 reads or 2% of the variant allele frequency supported the mutation; 2) if a population frequency >1% was present in the 1000 Genomes or ExAC databases. The final list of mutations was then annotated using vcf2maf. The mutation feature set for each patient displayed in the detection report is a simulation of the true mutation level that incorporates measurement errors. This enables machine learning models to better assess the undetectable risk of measurement that occurs in clinical practice. In total, we simulated 660 patients, and the TMB index for each patient was divided into 3 dimensions based on point mutations (SNVs), insertion mutations (INSs), and deletion mutations (DELs). Thus, $TMB={\left[SNV,INS,DEL\right]}^{T}$ . We note that chromosomal translocation, a type of structural abnormality, was not included in our experiments due to computational costs. However, our model is capable of handling additional forms of mutational burden, including copy number variations, microsatellite instability, and many others with neoantigenic potential. The input structure of the model is unaffected by mutation type.

Next, along with the mutation levels of the 660 simulated patients, we also simulated patient efficacy endpoints in response to immunotherapy. The ORR endpoint was generated based on a dichotomous logistic probability. In this paper, the probability of remission was directly retained to characterize the ORR endpoint efficacy. This was to facilitate the labeling of prognostic labels for patient subgroups. In contrast, progression-free survival records were generated based on the probability density function of Cox PH and corresponding mutational features. The specific simulation process was described in detail in previous papers (25, 26). The prognostic labels of patient subgroups with different treatment effects formed five subgroups containing 90, 212, 64, 202, and 92 patients, respectively. These groups had the following corresponding prognostic labels: (0.223,0246), (0.326,0397), (0.461,0.558), (0.654,0.733), and (0.908,0.922), respectively.

### 3.2 SYUCC patient information

For the SYUCC patient cohort, we retrospectively examined 64 patients with R/M NPC who were treated with anti-PD-(L)1 or anti-CTLA-4 (NCT02721589 and NCT02593786) as well as 73 patients with NSCLC who underwent anti-PD-(L)1 monotherapy. The trial design for the dosage escalation and expansion phases has been previously discussed (36–38). Patients eligible for enrollment were between the ages of 18 and 70, had histologically or cytologically verified locally advanced or metastatic NSCLC or NPC, an ECOG performance status score of 0 or 1, at least one RECIST 1.1 detectable lesion, and had failed at least one prior systemic therapy. Exclusion criteria included metastases to the central nervous system, prior malignancy, autoimmune disease, prior immunotherapy, active tuberculosis infection, pregnancy, or treatment with an immunosuppressive agent.

In this dataset, 60% of patients with lung cancer had adenocarcinoma, and 32% had squamous carcinoma. At the time of diagnosis, nearly all patients (99%) were in stage IV. The median age of patients with NSCLC and NPC at the start of treatment was 55 and 46 years, respectively. Smoking history was present in 49% of NSCLC patients and 25% of NPC patients, with more males than females in both cohorts (70% vs. 30% for NSCLC, 80% vs. 20% for NPC). Moreover, the ORR of the study cohorts was 19% and 12%, respectively, and the median PFS was 91 days for NSCLC and 67.5 days for NPC. The distribution of patient treatments, as well as the library preparation, sequencing, and bioinformatics procedures has been described in detail in a previous study (25).

Finally, the one-dimensional TMB index was subdivided into more precise high-dimensional vectors based on mutation type to better characterize the genomic mutations associated with immunotherapeutic mechanisms. TMB metrics of each patient were vectorized based on their BAM files and the annotated VCF files of SYUCC cohort patients were used as the input feature vectors of the patients for learning. Next, the efficacy data of different patient subgroups were used as the expected output of the training learner.

### 3.3 Nonlinear associations between genomic mutations and ICI prognoses

**Figure 2:**
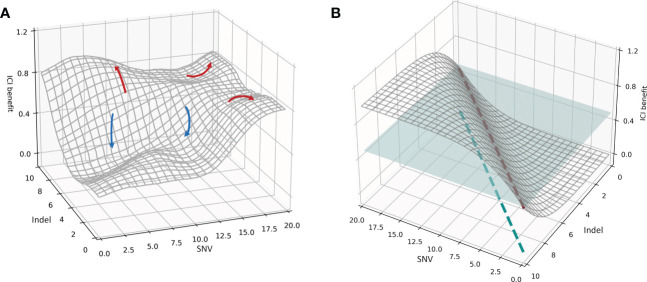
**(A)** Spatial distribution of the association between the mutational burdens and ICI composite prognosis. The red curve represents a favorable prognosis, while the blue curve represents an adverse prognosis. **(B)** Spatial distribution of the ideal association between mutational burdens and ICI composite prognosis. The blue plane represents a segmentation plane used to separate positive and negative prognoses. The red dashed line is the only intersection between the segmentation plane and the correlation distribution surface, and the blue dashed line indicates the projection of the red dashed line. The patient subgroup on the left of the blue dashed line is the population receiving superior results from immunotherapy.

The purpose of this paper is to mine the mechanisms responsible for the deep association between various mutation burdens and the overall efficacy of immunotherapy, to better understand the clinical benefit of ICI and to develop a decision framework using multidimensional biomarkers and multiscale endpoints in order to screen superior patient groups. Therefore, the general association distribution was fitted to investigate the mapping of decision biomarkers to efficacy endpoints. The actual spatial distribution between the multidimensional mutation burden and immunotherapy prognoses based on SYUCC cohort data, as well as the ideal spatial distribution, is shown in **Figure 2**.

Using the SYUCC cohort data, we then fitted the association distribution for two representative mutations, the SNV and Indel burdens, along with the comprehensive benefit of immunotherapy in three-dimensional space (**Figure 2**); we note that here the surface of the distribution in **Figure 2A** was Gaussian smoothed. **Figure 2A** demonstrates that when the Y-axis is held constant, and an increase in the SNV index is observed along the X-axis, patient ICI benefit rises. Similarly, when the X-axis is fixed, and the Indel burden (Y-axis) increases, the patient realizes a progressively more favorable prognosis. Both trends are compatible with the mechanisms by which immunotherapies induce antitumor immunogenicity. However, in real systems multiple peaks and valleys of ICI benefit exist, indicating that the correlation surface is not uniformly distributed in three-dimensional space. For example, the red arrows in **Figure 2A** indicate an improved prognosis for the patient, whereas blue arrows indicate a worsening prognosis. SNV-High and Indel-High conditions showed interdependencies across different dimensions, and the populations covered by both vary. In addition, we also found a discrepancy between multiple clinical efficacy endpoints, both in degree and direction. Consequently, several red arrows present in **Figure 2A**, imply that a specific subgroup of patients surrounding an ICI peak are encircled by their respective X- and Y-axes (i.e., these groups are jointly determined by SNV and Indel burdens at that location); these patients possess significantly better treatment outcomes. Likewise, multiple blue arrows indicate ICI troughs that reveal that patient subgroups lying within this range have subpar outcomes.

We then hypothesized an ideal condition in which the SNV and Indel burdens were independent of one another, and the direction and extent of the beneficial effect on ICI remain consistent. We simulated the distribution of this ideal association in three-dimensional space (**Figure 2B**). **Figure 2B** indicates that the therapeutic benefit of patients treated with ICI increases proportionally with the number of SNV and Indel mutations. Thus, the blue segmentation plane used to stratify efficacy and the association distribution surface have only a single intersection line, denoted by the red dashed line. This line was then mapped onto the X- and Y-axes, and the results suggest that the subgroup of patients on the left side of the blue dashed line is the superior group with respect to expected immunotherapeutic outcome. Moreover, a split line established using SNV and Indel measurements divides the cohort into two subgroups with significantly different efficacies.

However, there is more than one line of intersection between the tangent plane and the association distribution surface. This corresponds to the non-uniform distribution association surface in **Figure 2A**, and means that a single segmentation line defined by SNV and Indel metrics alone cannot divide the patient cohort into two subgroups with distinctly different efficacies. Thus, it is clear from **Figure 2A** that a complex nonlinear association exists between multidimensional mutations and immunotherapy prognosis. Furthermore it also indicates that multiple joint thresholds enable patients to form multiple subgroups with different treatment risks.

### 3.4 Analysis of prognostic predictions

First, to verify the effectiveness of the proposed multi-biomarker-multi-endpoint fusion decision, its prediction performance was assessed using simulated data consisting of 660 patients. These were divided into five subgroups and the input feature vectors were matched to output prognostic labels. The ORR&TTE prognostic labels for individual subgroups were defined using empirical labels and probabilistic labels based on the objective endpoints of patients, respectively. In addition, the subgroup efficacy measure and error calculation formula were gradually modified. The 10-fold cross-validation method was used to divide the simulation data into training and testing sets to avoid the risk of overestimating or underestimating the actual performance of the prediction model. Furthermore, in addition to using the cross-validation method, the best-performing model was selected for independent testing throughout the training phase to evaluate its prediction performance for unseen patients. Finally, the prediction and learning performance of the proposed TMBserval were measured using a distinct loss metric between the actual and the desired output, as well as the learning curve of machine learning.

As seen from the results in **Table 1**, when the prognosis labels of the multiple-instance learner were based on manually annotated category labels, overall prediction accuracy was poor, irrespective of whether the efficacy metric for the patient subgroup was taken as the maximum, minimum, median, or mean value. The calibration loss on the training set ranged from 0.1878 to 0.2811, while the calibration loss on the test set ranged from 0.2030 to 0.3333. Moreover, the calibration loss of the 10-fold cross-validation set ranged from 0.1954 to 0.3015. Among all metrics, the top performer was the mean-based efficacy metric, since mean-based calculations were essentially learned for each instance (i.e., for each patient), which allowed the learner to optimize based on all patients to a greater extent.

**Table 1:** Error results of simulation data under training set, independent testing set, and 10-fold cross-validation.

Prognostic Label	Prognostic Metrics	Training Error	Testing Error	10-fold Cross-validation Error
$y_{m}\in \{0,1\}$	Median	0.2811	0.3333	0.2924
$y_{m}\in \{0,1\}$	Maximum	0.2121	0.2895	0.3015
$y_{m}\in \{0,1\}$	Minimum	0.2693	0.3013	0.2969
$y_{m}\in \{0,1\}$	Mean	0.1878	0.2030	0.1954
$y_{m}=[p_{R},p_{T}]$	Median	0.1591	0.2255	0.2020
$y_{m}=[p_{R},p_{T}]$	Maximum	0.0222	0.0564	0.0329
$y_{m}=[p_{R},p_{T}]$	Minimum	0.1065	0.1544	0.1674
$y_{m}=[p_{R},p_{T}]$	Mean	0.0135	0.0060	0.0184

When the prognostic labels were probabilistically labeled based on objective endpoint observations for individual patients, the predictive performance of the MIL learner was greatly improved, showing a minimum Euclidean distance loss of 0.0135 and a maximum of 0.1591 for the training set, a minimum Euclidean distance loss of 0.0060 and a maximum of 0.2255 for the test set, and a minimum Euclidean distance loss for the 10-fold cross-validation set. All Euclidean distance losses were significantly less than calibration losses under category labels. This shows that manual label identification based on physician experience is somewhat irrational, and results in prognostic labels that do not accurately reflect the actual treatment outcomes of patients. This impedes the learner from establishing a proper mapping relationship and produces wildly inaccurate predictions for unseen patients. In contrast, when the prognostic labels were annotated according to the probability of objective endpoint observations, the overall prediction performance of the learner is greatly enhanced *via* better output feature characterization.

In conclusion, the simulation data prediction findings shown in **Table 1** indicate that the proposed multi-biomarker-multi-endpoint fusion MIL learner is able to accomplish the prediction task for unseen patients/patient subgroups effectively and can formulate thorough and accurate evaluations. On the other hand, proposed networks with calibration loss as the optimization target not only ensures the accuracy of patient categorization, but also highlights the statistical interpretability of the model in terms of goodness-of-fit compared to conventional loss measurements. In this paper, we further compare the support vector machine (SVM), the mean square error (MSE) measure, and the cross-entropy loss (CEL) measure, which are common in machine learning, with the calibration loss metric calculated using TMBserval. **Table 2** compares the differences in calibration performance of these different models using a Hosmer-Lemeshow test.

**Table 2:** Comparison of goodness-of-fit tests based on different error metric learners.

Data set	HL statistics under Calibration Error metrics	*p*-value of goodness-of-fit test	HL statistics under CEL metrics	*p*-value of goodness-of-fit test
Training set	**HL-chi2(8): 8.85**	**0.3553**	HL-chi2(8): 12.282	0.1390
Testing set	**HL-chi2(8): 10.15**	**0.2548**	HL-chi2(8): 20.276	0.0093
All	**HL-chi2(8): 7.54**	**0.4797**	HL-chi2(8): 16.113	0.0407
Data set	HL statistics under MSE metrics	*p*-value of goodness-of-fit test	HL statistics under SVM	*p*-value of goodness-of-fit test
Training set	HL-chi2(8): 14.232	0.0759	HL-chi2(8): 11.6310	0.1684
Testing set	HL-chi2(8): 18.623	0.0170	HL-chi2(8): 15.3936	0.0519
All	HL-chi2(8): 11.226	0.1892	HL-chi2(8): 9.799	0.2793
Data set	HL statistics under Calibration Error metrics	*p*-value of goodness-of-fit test	HL statistics under CEL metrics	*p*-value of goodness-of-fit test
Training set	**HL-chi2(18): 19.407**	**0.3671**	HL-chi2(18): 23.193	0.1832
Testing set	**HL-chi2(18): 12.724**	**0.8076**	HL-chi2(18): 14.843	0.6727
All	**HL-chi2(18): 20.734**	**0.2930**	HL-chi2(18): 24.257	0.1467
Data set	HL statistics under MSE metrics	*p*-value of goodness-of-fit test	HL statistics under SVM	*p*-value of goodness-of-fit test
Training set	HL-chi2(18): 23.101	0.1867	HL-chi2(18): 23.329	0.1782
Testing set	HL-chi2(18): 14.785	0.6766	HL-chi2(18): 26.079	0.0979
All	HL-chi2(18): 28.668	0.0525	HL-chi2(18): 27.669	0.0672

Bold values indicate the best performance in the HL tests.

For the Hosmer-Lemeshow test, we varied the degrees of freedom (i.e., 10 versus 20 groups), to explore the goodness-of-fit performances of different learners. As seen from the results shown in **Table 2**, a typical MIL learner using the CEL metric as the loss function exhibited the worst fit under 8 degrees of freedom, with a *p*-value of 0.1390 for the training set, a *p*-value of 0.0093 for the testing set, and a *p*-value of 0.0407 for the whole set. These values indicate that the learner does not show an excellent goodness-of-fit. In contrast, the calibration loss metric proposed in this paper considers the goodness-of-fit of the model while ensuring the prediction performance of the learner. This demonstrates superior results in the Hosmer-Lemeshow test, with *p*-values of 0.3553 and 0.4797 for the training and complete sets, respectively, and a *p*-value of 0.2548 for the independent testing set. Likewise, TMBserval remained the best performer among the four types of learners in a scenario with 18 degrees of freedom, with *p*-values of 0.3671 and 0.2930 for the training and full sets, respectively, and a *p*-value of 0.8076 for the independent testing set. The dominance of TMBserval in the HL test further demonstrates that the calibration loss proposed by this paper significantly increases the learner’s statistical interpretability.

**Figure 3:**
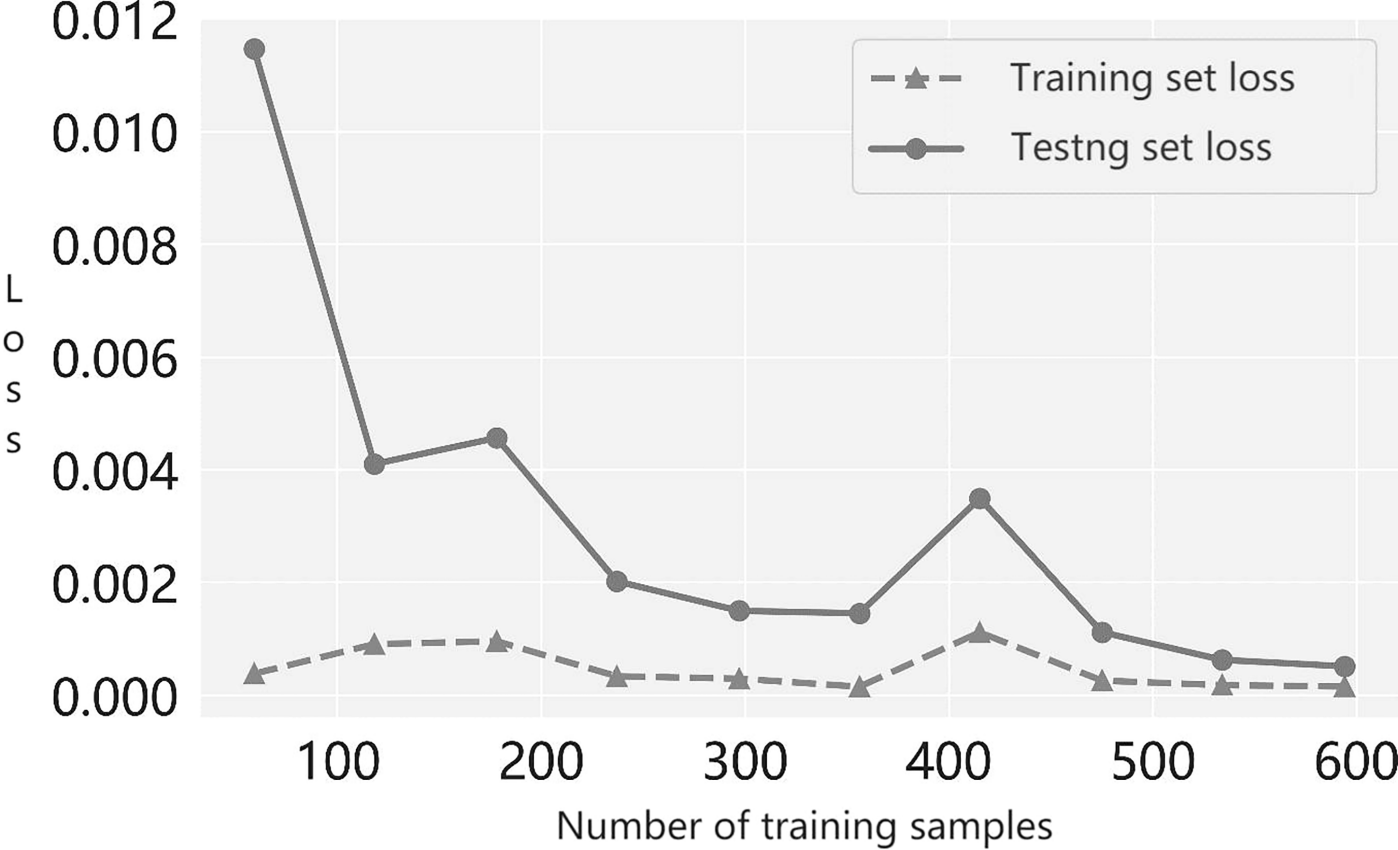
The learning curve of the prediction model.

Next, to determine whether the suggested multiple-instance learner under- or overfits models, we plotted the prediction accuracy of the modeled learner with the number of training samples; this is the learning curve. From the trend of the loss curve shown in **Figure 3**, we see that the loss in the cross-validation set steadily decreased and converged as more training samples were incorporated. Moreover, the gap between the loss of the training set and the loss of the validation set narrowed. Therefore, our results show that the learner proposed in this paper neither overfits nor underfits models during the training process.

In addition, this paper establishes a many-to-many mapping relationship based on a real SYUCC experimental cohort (i.e., consisting of 137 patients from three patient subgroups) from the input TMB feature vector for each patient to corresponding output subgroup prognostic labels. This was done to evaluate the predictive accuracy of TMBserval for unseen patients and/or subgroups. Similarly, the comprehensive ORR&TTE prognostic labels for patient subgroups were labeled using empirical and objective-based probabilistic methods, respectively. The predictive performance of the proposed model was evaluated by varying subgroup efficacy metrics as well as the loss calculation formulas. This was done using 10-fold cross-validation as well as independent test validation. Ultimately, the usefulness of the proposed model for clinical practice was measured by the loss between the actual and the expected output.

As summarized in **Table 3**, the prediction performance of the multi-biomarker-multi-endpoint fusion MIL learner remained accurate regardless of the labeling approach upon which it was built, albeit with some variability among different metrics. For example, for category-based prognostic labels, when the efficacy metric for patient subgroups was taken as the minimum, the prediction loss of the learner was significantly higher than for the other models, with a calibration loss of 0.0294 for the training set, 0.1052 for the testing set, and 0.0806 for the 10-fold cross-validation. Thus, the criteria used to assess the efficacy of patient subgroups based on minimum values do not appear to be applicable for the NSCLC and nasopharyngeal carcinoma subtypes included in the SYUCC patient cohort in this paper.

**Table 3:** Error results for patient data under training set, independent test set and 10-fold cross validation.

Prognostic Label	Prognostic Metrics	Training Error	Testing Error	10-fold Cross-validation Error
$y_{m}\in \{0,1\}$	Median	0.0145	0.0526	0.0640
$y_{m}\in \{0,1\}$	Maximum	0.0117	0.0426	0.0741
$y_{m}\in \{0,1\}$	Minimum	0.0294	0.1052	0.0821
$y_{m}\in \{0,1\}$	Mean	0.0058	0.0117	0.0587
$y_{m}=[p_{R},p_{T}]$	Median	0.0132	0.0015	0.0143
$y_{m}=[p_{R},p_{T}]$	Maximum	0.0103	0.0135	0.0157
$y_{m}=[p_{R},p_{T}]$	Minimum	0.0030	0.0022	0.0064
$y_{m}=[p_{R},p_{T}]$	Mean	0.0378	0.0224	0.0390

When probabilistic labeling of prognostic labels was then performed using the objective endpoint observations for patients, the multi-instance learner’s prediction performance improved moderately. The prediction loss remained remarkably low, and still marginally outperformed the category label-based learner. Manual annotation methods based on physician experience have limitations for clinical practice due to their subjective nature. Based on pooled analysis results, this paper suggests using the latter to obtain prognostic labels for clinical practice, and this is more conducive to the promotion and application of the learner.

Similarly, we conducted comparison experiments using the SYUCC cohort to verify the goodness-of-fit of the model. As shown in **Table 4**, for the SYUCC experimental cohort, the calibration loss model proposed in this paper maintains a superior goodness-of-fit than the traditional CEL metric, MSE metric, or SVM classifier, regardless of whether the degree of freedom was 8 or 18. This was evident since the Hosmer-Lemeshow test results were outstanding. Under 8 degrees of freedom, the *p*-values of HL goodness-of-fit for the training set, testing set, and full set were 0.9789, 0.7692, and 0.7692, respectively. However, under 18 degrees of freedom, the *p*-values of the HL goodness-of-fit for the training set, testing set, and full set were 0.9369, 0.9948 and 0.7843, respectively. Taken together, these results show that the proposed fusion decision model not only can distinguish patients correctly but can also ensures the consistency between the actual probability of occurrence of a particular outcome and its probability as predicted by the model.

**Table 4:** Comparison of goodness-of-fit tests based on different error metric learners.

Data set	HL statistics under Calibration Error metrics	*p*-value of goodness-of-fit test	HL statistics under CEL metrics	*p*-value of goodness-of-fit test
Training set	**HL-chi2(8): 2.065**	**0.9789**	HL-chi2(8): 11.008	0.2012
Testing set	**HL-chi2(8): 4.88**	**0.7692**	HL-chi2(8): 8.518	0.3846
All	**HL-chi2(8): 4.89**	**0.7692**	HL-chi2(8): 8.289	0.4057
Data set	HL statistics under MSE metrics	*p*-value of goodness-of-fit test	HL statistics under SVM	*p*-value of goodness-of-fit test
Training set	HL-chi2(8): 11.918	0.1549	HL-chi2(8): 16.537	0.0353
Testing set	HL-chi2(8): 8.967	0.3451	HL-chi2(8): 12.693	0.1228
All	HL-chi2(8): 12.415	0.1336	HL-chi2(8): 13.647	0.0914
Data set	HL statistics under Calibration Error metrics	*p*-value of goodness-of-fit test	HL statistics under CEL metrics	p-value of goodness-of-fit test
Training set	**HL-chi2(18): 9.841**	**0.9369**	HL-chi2(18): 20.925	0.2832
Testing set	**HL-chi2(18): 6.287**	**0.9948**	HL-chi2(18): 15.955	0.5957
All	**HL-chi2(18): 13.122**	**0.7843**	HL-chi2(18): 25.548	0.1105
Data set	HL statistics under MSE metrics	*p*-value of goodness-of-fit test	HL statistics under SVM	*p*-value of goodness-of-fit test
Training set	HL-chi2(18): 17.211	0.5086	HL-chi2(18): 28.631	0.0531
Testing set	HL-chi2(18): 7.614	0.9837	HL-chi2(18): 13.736	0.7461
All	HL-chi2(18): 21.306	0.2642	HL-chi2(18): 24.442	0.1411

Bold values indicate the best performance in the HL tests.

## 4 Discussion

In clinical practice, relying only on a single predictive biomarker and a single observed endpoint of efficacy to guide immunotherapy is inadequate. Moreover, the one-dimensional TMB metric suffers from challenges because it equally quantifies all types of mutations. The trend toward higher dimensional vectorization of TMB is inevitable. Synergistic analysis of multidimensional mutation burdens can provide a stronger predictive value for patient outcomes. However, current clinical decision-supporting models are primarily used to identify and apply single predictive markers, and most decision endpoints rely on a single observation, thereby creating a one-to-one mapping relationship. Thus, there is a lack of available, reasonable, and rigorous modeling methods to solve the many-to-many problem. ICI multiscale endpoints must rely on decision-making based on multidimensional synergistic biomarkers. In the face of complex interdependencies between biomarkers and the associations between decision endpoints, traditional mathematical methods cannot establish a conforming nonlinear mapping relationship between non-independent inputs and outputs. At the same time, the ultimate optimization goal of this paper is a form of categorical decision-making; therefore, prognostic labels are based on the description of disease treatment effects at the patient subgroup level. Therefore, to resolve the computational difficulties listed above, this paper establishes a nonlinear mapping model implementing multi-biomarker to multi-endpoint data that uses an MIL framework; this model is called TMBserval **(**i.e., Statistical Explainable machine learning with Regression-based VALidation).

In this paper, we subdivided the one-dimensional TMB index into more precise high-dimensional vectors based on mutation type to better characterize the genomic mutations that were associated with immunotherapy mechanisms. Some investigators have found that the mutations that are unlikely to be lost under the selective pressure of immunotherapy were strongly associated with immunotherapy benefits. TMB can be segmented not only by mutation type but also by dynamic changes. Therefore, our next stage will be to consider longitudinal measurement of mutational characteristics and clinical treatment outcomes in a cohort of patients. We will then develop a dynamic decision-making model based on time-series biomarkers to provide more accurate prognoses for the clinical practice of tumor immunotherapy.

By revising the definition of the loss function during the training of the ideal nonlinear mapping, this paper establishes a statistically interpretable loss metric that considers the clinical characteristics of immunotherapy and establishes different metrics for experience-based category labels and objective-based probabilistic labels, respectively. The consistency of the proposed model for absolute risk prediction is ensured by defining a loss measure function that considers the calibration of the model, while the ability of the proposed model to discriminate between patient cohorts is ensured by reasonably defining prognostic labels for different patient subgroups. In addition, the correlation between the ORR and TTE endpoints was incorporated into the model optimization objective *via* considering the Mahalanobis distance. Back propagation based on the gradient descent method minimizes the loss function and optimizes the regression network to reach the training target. This eventually results in a nonlinear mapping model with wide applicability, which can make correct predictions for unseen patients or patient subgroups.

## 5 Conclusion

TMBserval 1) solved the problem that multidimensional predictive mutations and multiscale efficacy observation endpoints are difficult to combine and analyze together to build a pan-cancer-oriented many-to-many nonlinear regression model with reasonable mapping accuracy; 2) formed a categorical decision-making model for ICI, built a regression model based on immunotherapeutic results on the patient subgroup level to obtain joint thresholds for multi-categorization and to distinguish more finely and accurately between patient groups in a high-dimensional feature space; 3) constructed a learner according to the standard statistical criteria for discrimination and calibration measures, rendering the trained model more statistically interpretable and ameliorating the black-box characteristics of machine learning, thereby making it more accessible to clinicians. We conclude that TMBserval can enhance precision immunotherapy decision-making and expand the population of patients that can benefit from immunotherapy.

## Data availability statement

The original contributions presented in the study are included in the article/**Supplementary Material**. Further inquiries can be directed to the corresponding authors.

## Ethics statement

The studies involving human participants were reviewed and approved by Sun Yat-sen University Cancer Center’s Ethical Review Committee. The patients/participants provided their written informed consent to participate in this study.

## Author contributions

YW, YL, XL, and XS conceived and designed the study. YW, JW, and WF developed the methodology. YW, JW, WF, SY and XX collected and managed the data. YW wrote the first draft. YW, JW, QW, YL, XL, and XS reviewed, edited, and approved the manuscript. JW, XX, QW, JZ, JL, and WF provided administrative, technical, or material support. JW, and XS were primarily responsible for the final manuscript. All authors contributed to the article and approved the submitted version.
